# Clinical and Echocardiographic Risk Factors of Adverse Outcomes in Young Patients With Dilated Cardiomyopathy

**DOI:** 10.1155/cdr/2122089

**Published:** 2025-03-08

**Authors:** Mengwan Li, Wenheng Liu, Shouling Mi, Meng Wang, Yanli Wang, Qilong Guo, Zhexun Lian, Junhua Ge

**Affiliations:** ^1^Department of Cardiology, The Affiliated Hospital of Qingdao University, Qingdao, Shandong Province, China; ^2^Qingdao Municipal Key Laboratory of Hypertension (Key Laboratory of Cardiovascular Medicine), Qingdao, Shandong Province, China; ^3^Department of Cardiology, Zhongshan Hospital, Fudan University, Shanghai, China; ^4^Shanghai Institute of Cardiovascular Diseases, Shanghai, China; ^5^State Key Laboratory of Cardiovascular Diseases, Shanghai, China; ^6^NHC Key Laboratory of Ischemic Heart Diseases, Shanghai, China; ^7^Key Laboratory of Viral Heart Diseases, Chinese Academy of Medical Sciences, Shanghai, China; ^8^National Clinical Research Center for Interventional Medicine, Shanghai, China

**Keywords:** dilated cardiomyopathy, outcome, risk factors, young adults

## Abstract

**Purpose:** This study is aimed at identifying clinical and echocardiographic factors associated with all-cause mortality or heart transplantation (HTx) in young patients with dilated cardiomyopathy (DCM).

**Methods:** We conducted a retrospective analysis of hospitalized patients (aged 18–45 years) diagnosed with DCM between January 2012 and December 2022. All patients underwent a 2-year medical therapy for heart failure, followed by at least 1 year of follow-up. Clinical and echocardiographic data were collected at baseline and after the 2-year treatment period. Multivariate Cox proportional hazards regression with a backward stepwise method was used to identify risk factors for all-cause mortality or HTx.

**Results:** The study cohort comprised 67 patients. Over a median follow-up of 38 months (range 18–50), 15 patients died and 24 underwent HTx. Significant risk factors for all-cause mortality/HTx included smoking, digoxin use, elevated N-terminal pro-brain natriuretic peptide (NT-proBNP, ≥ 5678 pg/mL), higher C-reactive protein (CRP, ≥ 3.0 mg/L), higher uric acid (UA, ≥ 570 *μ*mol/L), lower left ventricular ejection fraction (LVEF, ≤25%), and enlarged end-diastolic left ventricular diameter (LVD, ≥ 65 mm). Among these, elevated CRP (hazard ratio, HR = 6.727, *p* < 0.001) and enlarged LVD (HR = 3.038, *p* = 0.007) were the strongest independent risk factors, irrespective of other risk factors. Moreover, each 5 mm annual increase in end-systolic left atrial diameter (LAD, HR = 3.641, *p* < 0.001) and each unit annual increase in Ln(NT-proBNP) (HR = 4.069, *p* < 0.001) were the strongest predictors of all-cause mortality/HTx, even after accounting for the effects of body mass index, duration of treatment, and baseline CRP level.

**Conclusions:** Intensive monitoring and medical care may be beneficial for young adult DCM patients with defined risk factors such as smoking, elevated NT-proBNP and CRP, lower LVEF, and enlarged LV diameter. Our findings suggest that personalized intensive monitoring and medical care based on identified risk factors may improve outcomes in young adult DCM patients.

## 1. Introduction

Dilated cardiomyopathy (DCM) is characterized by left ventricular (LV) chamber enlargement and systolic dysfunction without identifiable abnormal loading conditions or significant coronary artery disease [1]. Progressive ventricular enlargement leads to ventricular dysfunction, conduction abnormalities, arrhythmias, thromboembolism, and heart failure, culminating in adverse clinical outcomes [2, 3]. DCM ranks as the third most common form of heart failure and the leading cause of heart transplantation (HTx). It affects approximately 1 in 2500 individuals [4]. The etiology of DCM is diverse, encompassing both genetic and environmental factors. Primary DCM arises from pathogenic gene variants, while secondary DCM results from acquired factors such as infections, autoimmunity, toxins (e.g., alcohol, recreational drugs, or cancer therapy), endocrinopathies, and tachyarrhythmias [1].

Assessment of LV enlargement and LV dysfunction remains central to diagnosis, risk stratification, and decision-making for treatment. Beyond these parameters, there might be other clinical indexes with clinical importance in terms of prognosis and significant therapeutic implications. In fact, advanced imaging techniques [5], together with modern genetic testing [6], blood biomarkers [7], and biopsy analysis [8], are more frequently used in the clinical assessment of DCM patients. Indexes derived from these analyses might serve as additional prognostic predictors. Several pediatric DCM cohorts have identified risk factors for adverse outcomes, including older age (> 6 years), lower LV fractional shortening, heart failure at presentation, idiopathic DCM, and elevated N-terminal pro-brain natriuretic peptide (NT-proBNP) levels. Early risk stratification and targeted interventions are crucial to improving prognosis in high-risk patients [3, 9].

Young adults represent a distinct demographic among patients with DCM, with their disease progression and outcomes potentially differing from those of older patients. Identifying risk factors associated with adverse outcomes in young individuals with DCM is crucial for effective risk assessment and personalized treatment strategies. Despite extensive research on older adults with DCM, there remains a significant knowledge gap regarding outcome predictors for young patients. Our study addresses this gap by analyzing clinical and echocardiographic data to identify independent risk factors for adverse outcomes in young DCM patients aged 18–45 years. The aim is to improve risk stratification and provide empirical support for tailoring interventions to individual patient needs.

## 2. Methods

### 2.1. Ethics Approval

The research adhered to the principles outlined in the Declaration of Helsinki. Approval for the study protocol was obtained from the Ethics Committee of the Affiliated Hospital of Qingdao University (Ethics Number: QYFY WZLL 28022; decision date: August 17, 2023). Informed consent was obtained from all participants, either directly from subjects or through legally authorized representatives, through both verbal and written communication.

### 2.2. Study Population

We conducted a retrospective cohort study that screened and included hospitalized patients aged 18–45 years with a first-time diagnosis of DCM in our hospital between January 2012 and December 2022. The diagnosis of DCM involved a comprehensive assessment approach, including considering family history to evaluate genetic predisposition and environmental factors, reviewing clinical history, conducting physical examinations, analyzing blood investigations, and utilizing various imaging modalities such as radiography and echocardiography [10, 11]. The diagnostic criteria for DCM included (1) echocardiography-detected LV dilatation, accompanied by reduced LV systolic function (left ventricular ejection fraction, LVEF < 45%); (2) exclusion of angiographic or valvular causes of LV dilatation and dysfunction; and (3) exclusion of other potential contributors to systolic dysfunction, such as alcohol abuse, chemotoxicity, congenital heart disease, neuromuscular disease, or systemic diseases. All enrolled patients received standard medical treatment for heart failure in accordance with respective guidelines [12].

### 2.3. Data Collection and Definitions

Data were collected by trained medical personnel, including clinicians and research assistants, under the supervision of experienced investigators (J.G. and M.L.). All collected data underwent verification for accuracy. Data collection occurred at two time points: initially at baseline (admission) and subsequently after the 2-year medical therapy period for heart failure. At baseline, comprehensive data including demographic information, comorbidities, cardiac risk factors, laboratory results, medication profiles, and echocardiographic findings were recorded. Following the 2-year medical therapy period, follow-up data were gathered to monitor longitudinal changes. This included repeated measurements of echocardiographic parameters and NT-proBNP levels.

Chronic kidney disease was diagnosed based on an estimated glomerular filtration rate (eGFR) of less than 60 mL/min/1.73 m^2^ for at least 3 months and/or the presence of kidney damage, such as albuminuria, for at least 3 months [13]. Smoking and drinking status was determined by inquiring about the patient's current habits [14]. Drinking quantity was defined as consuming more than 30 g on any single day and more than 70 g/week for women, and more than 40 g on any single day and more than 140 g/week for men.

### 2.4. Outcomes

Patients were followed for a minimum of 1 year after completing the 2-year medical therapy period. The primary endpoint was defined as all-cause mortality or HTx. Follow-up data were collected through medical record reviews, regular clinical visits, and telephone interviews.

### 2.5. Echocardiography

Echocardiographic parameters were measured offline according to the current guidelines [15]. LVEF was measured using the Simpson biplane method in LV apical four- and two-chamber views. End-diastolic left ventricular diameter (LVD) and end-systolic left atrial diameter (LAD) were measured in an LV long-axis view. End-diastolic right ventricular mid diameter (RVD), end-systolic right atrial long-axis diameter (RAD_long), and end-systolic right atrial short-axis diameter (RAD_short) were measured in an RV-focused apical four-chamber view.

### 2.6. Statistical Analysis

Continuous variables are presented as mean ± standard deviation or median and interquartile range (IQR), while categorical variables are expressed as numbers (percentage). For normally distributed continuous variables, we used the independent sample *T*-test; for non-normally distributed variables, we employed the Mann–Whitney *U* test. Categorical data were compared using the Chi-square test or Fisher's exact test, as appropriate.

Optimal cut-offs for potential risk variables associated with all-cause mortality/HTx were determined using the Youden index method derived from receiver operating characteristic (ROC) curves. The Kaplan–Meier curves and log-rank tests were utilized to compare cumulative survival probabilities between groups based on these risk variables.

We performed both univariable and multivariable Cox proportional hazards regression analyses to identify risk factors associated with outcomes. Two distinct follow-up periods were used in the Cox models: (1) for analyzing baseline variables associated with all-cause mortality/HTx risk: the follow-up period spanned from the baseline visit to the endpoint or the last day of follow-up, and (2) for assessing changes in variables over the 2-year medical therapy in relation to all-cause mortality/HTx: the follow-up period spanned from the completion of the 2-year medical therapy to the endpoint or the last day of follow-up. Hazard ratios (HRs) and 95% confidence intervals (CIs) were calculated to quantify the strength of associations. In the multivariable Cox regression model, potential risk factors identified through univariable analysis (*p* < 0.10) were adjusted for age, sex, body mass index (BMI), etiology of DCM, and duration of treatment using a backward stepwise (likelihood ratio) method. Additionally, comprehensive multivariable Cox regression models that included all potential factors, along with basic confounders such as age, sex, BMI, etiology of DCM, and duration of treatment, were used to identify the strongest risk factors associated with all-cause mortality/HTx.

A *p* value < 0.05 (two-tailed test) was considered statistically significant. All statistical analyses were performed using SPSS software, Version 23.0 (IBM SPSS Statistics, Chicago, United States).

We would like to disclose that the production of the submitted work did not involve the utilization of artificial intelligence (AI)–assisted technologies, such as large language models (LLMs), chatbots, or image creators.

## 3. Results

### 3.1. Outcomes and Groupings

**Figure 1:**
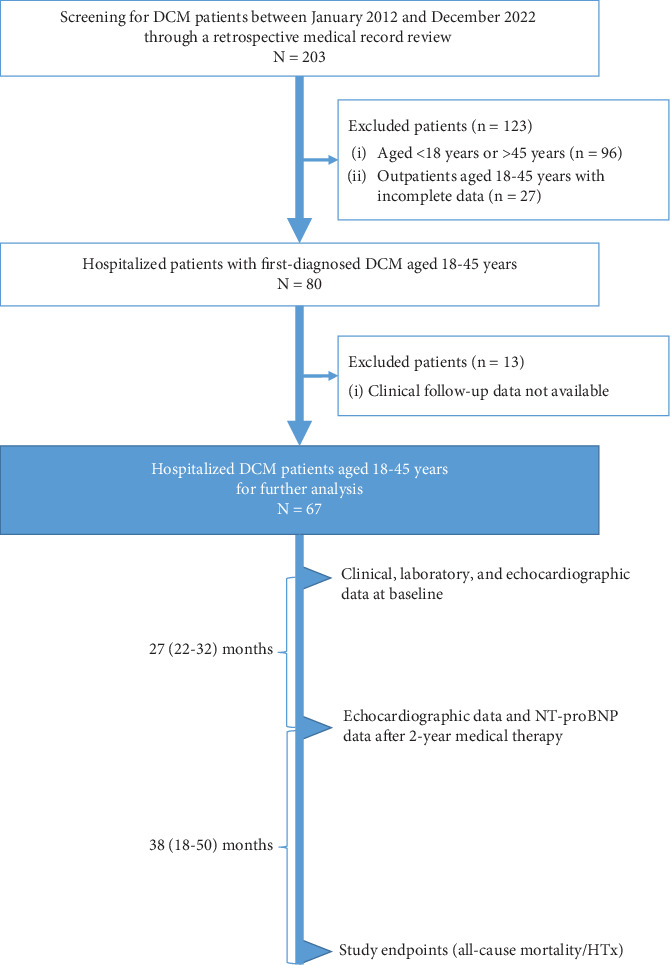
Study flowchart. DCM, dilated cardiomyopathy; HTx, heart transplantation; NT-proBNP, N-terminal pro-brain natriuretic peptide.

This retrospective study enrolled 67 patients (Figure 1). Initially, 203 patients diagnosed with DCM were screened through a retrospective review of medical records. Among them, 80 hospitalized patients, aged 18–45 years, with newly diagnosed DCM were selected. Thirteen patients were further excluded due to a lack of available clinical follow-up data. The etiologies of DCM among the enrolled patients were as follows: 15 with a genetic predisposition, 13 with myocarditis, two with inflammatory or infectious causes, three with exposure to toxic substances, and three with pregnancy-related DCM. The remaining patients (*n* = 31), with no identifiable etiology, were classified as idiopathic. None of these patients died during the initial 2-year medical therapy period postdischarge (median duration 27 months, IQR 22–32). Following the completion of the 2-year medical therapy, all patients underwent a median follow-up duration of 38 months (IQR 18–50). During the follow-up period, 15 patients died and 24 underwent HTx, forming the nonsurvivors/HTx group.

### 3.2. Baseline Characteristics Associated With All-Cause Mortality/HTx

The mean age at diagnosis of the included DCM patients was 31 ± 6 years, and 74.6% were male. Baseline clinical characteristics are summarised in Table 1. Compared to the survivor group, patients in the nonsurvivors/HTx group exhibited significantly lower BMI (24.7 ± 5.3 vs. 29.5 ± 6.0 kg/m^2^, *p* = 0.001), lower proportion of sacubitril/valsartan use (20.5% vs. 46.4%, *p* = 0.024), and lower LVEF (25.1% ± 5.1% vs. 30.3% ± 5.6%, *p* < 0.001). Conversely, the prevalence of smoking (46.2% vs. 10.7%, *p* = 0.006) and digoxin use (82.1% vs. 32.1%, *p* < 0.001) was significantly higher. Serum levels of NT-proBNP (median 9509 vs. 2321 pg/mL, *p* < 0.001), C-reactive protein (CRP, 6.00 vs. 1.20, *p* < 0.001), uric acid (UA, 682 vs. 523 *μ*mol/L, *p* = 0.012), LVD (68.8 ± 6.5 vs. 64.3 ± 4.7 mm, *p* = 0.003), and RAD_long (57.7 ± 8.5 vs. 53.3 ± 9.3 mm, *p* = 0.050) were significantly higher in the nonsurvivors/HTx group.

**Figure 2:**
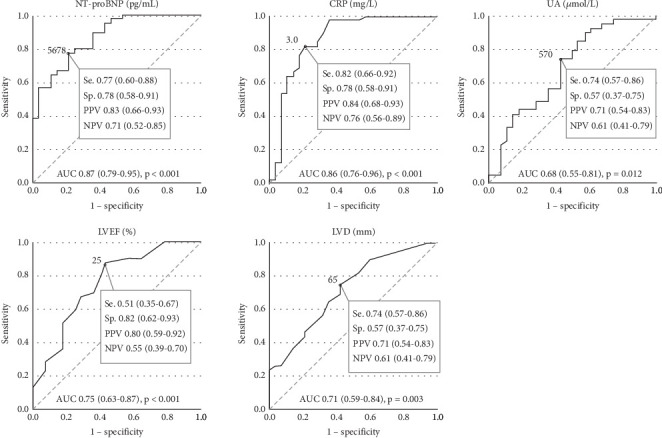
Receiver operating characteristic curves of baseline serum NT-proBNP, CRP, UA levels, LVEF, and LVD for predicting all-cause mortality/HTx in young patients with DCM. AUC, area under the receiver operating characteristic curve; CRP, C-reactive protein; DCM, dilated cardiomyopathy; HTx, heart transplantation; LVD, end-diastolic left ventricular diameter; LVEF, left ventricular ejection fraction; NPV, negative predictive value; NT-proBNP, N-terminal pro-brain natriuretic peptide; PPV, positive predictive value; Se., sensitivity; Sp., specificity; UA, uric acid.

**Figure 3:**
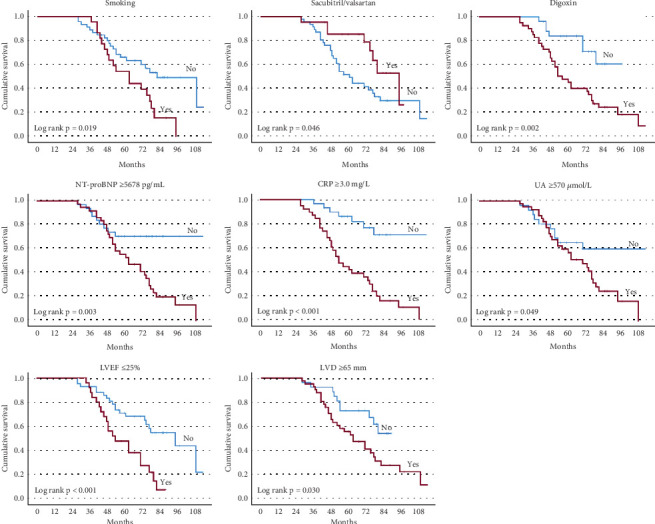
The Kaplan–Meier curves of baseline risk factors for predicting all-cause mortality/HTx in young patients with DCM. AUC, area under the receiver operating characteristic curve; CRP, C-reactive protein; DCM, dilated cardiomyopathy; HTx, heart transplantation; LVD, end-diastolic left ventricular diameter; LVEF, left ventricular ejection fraction; NT-proBNP, N-terminal pro-brain natriuretic peptide; UA, uric acid.

As shown in Table 2, univariable Cox regression analysis identified the following potential risk factors associated with an increased risk of all-cause mortality/HTx: lower BMI (HR 0.905, *p* = 0.001), smoking (HR 2.101, *p* = 0.023), absence of sacubitril/valsartan use (HR 0.463, *p* = 0.053), digoxin use (HR 3.294, *p* = 0.005), elevated NT-proBNP (≥ 5678 pg/mL, HR 2.876, *p* = 0.005), CRP (≥ 3.0 mg/L, HR 5.051, *p* < 0.001), UA (≥ 570 *μ*mol/L HR 2.020, *p* = 0.056) levels, lower LVEF (≤ 25%, HR 2.738, *p* = 0.003), and higher LVD (≥ 65 mm, HR 2.179, *p* = 0.036).  Figure 2 depicts the diagnostic efficacy of serum NT-proBNP, CRP, UA levels, LVEF, and LVD for predicting all-cause mortality/HTx, and their optimal cut-off values were identified. The Kaplan–Meier curves illustrated that patients with smoking, digoxin use, absence of sacubitril/valsartan use, NT-proBNP ≥ 5678 pg/mL, CRP ≥ 3.0 mg/L, UA ≥ 570 *μ*mol/L, LVEF ≤ 25%, and LVD ≥ 65 mm exhibited a significantly higher risk of all-cause mortality/HTx (Figure 3).

### 3.3. Dynamic Change in Cardiac Function and Chambers and NT-proBNP Following 2-Year Treatment and Their Prognostic Performance for All-Cause Mortality/HTx

As shown in Table 3, in the survivor group, there was a significant increase in LVEF from a median of 31%–39% (*p* < 0.001), while LVEF remained unchanged in the nonsurvivors/HTx group (median from 25%–23%, *p* = 0.266). All cardiac chambers, including LVD, RVD, LAD, RAD_long, and RAD_short, exhibited further dilation in the nonsurvivors/HTx group (all *p* < 0.05). In contrast, LVD reduced from 63 to 60 mm (*p* = 0.007), and RAD_long (from 53 to 48 mm, *p* = 0.015) and RAD_short (from 41 to 39 mm, *p* = 0.014) decreased in the survivor group.

The Cox regression analysis revealed that a decrease in LVEF over time (each 1% annual decrease, HR 1.088, *p* = 0.004), an increase in LVD (each 1 mm annual increase, HR 1.229, *p* < 0.001), an increase in LAD (each 1 mm annual increase, HR 1.077, *p* = 0.037), and increases in RAD_long (each 1 mm annual increase, HR 1.087, *p* = 0.009) and RAD_short (each 1 mm annual increase, HR 1.172, *p* = 0.002) over time were all found to be significantly associated with heightened risks of all-cause mortality/HTx.

During the course of treatment follow-up, NT-proBNP levels exhibited a decrease in both the survivor group (median decrease from 2321 to 861 pg/mL, *p* < 0.001) and the nonsurvivors/HTx group (median decrease from 9509 to 7068 pg/mL, *p* = 0.018). Notably, the reduction in NT-proBNP levels was more pronounced in the survivor group (median decrease of 1024 pg/mL) compared to the nonsurvivors/HTx group (median decrease of 862 pg/mL, *p* < 0.001). Elevated NT-proBNP levels during treatment follow-up were associated with a higher risk of all-cause mortality or HTx (each unit annual increase in Ln(NT-proBNP), HR 2.896, *p* < 0.001).

### 3.4. Independent Baseline Risk Factors Associated With All-Cause Mortality/HTx

Independent baseline risk factors associated with all-cause mortality/HTx in young adult patients with DCM included smoking (HR 2.339, *p* =0.008), digoxin use (HR 2.535, *p* = 0.031), elevated levels of NT-proBNP (≥ 5678 pg/mL, HR 2.284, *p* = 0.032), CRP (≥ 3.0 mg/L, HR 5.654, *p* < 0.001), UA (≥ 570 *μ*mol/L, HR 1.928, *p* = 0.074), lower LVEF (≤ 25%, HR 2.370, *p* = 0.011), and larger LVD (≥ 65 mm, HR 2.110, *p* = 0.045). These factors were identified after adjusting for age, sex, BMI, etiology of DCM, and duration of treatment (Table 4).

A multivariable Cox regression model that included these potential factors, along with basic confounders such as age, sex, BMI, etiology of DCM, and duration of treatment, demonstrated that elevated CRP (HR 6.727, *p* < 0.001) and enlarged LVD (HR 3.038, *p* = 0.007) were the strongest risk factors associated with all-cause mortality/HTx, irrespective of sex (male vs. female, HR 0.320, *p* = 0.010), BMI (HR 0.904, *p* = 0.002), smoking (HR 1.915, *p* = 0.094), and LVEF (HR 1.931, *p* = 0.074).

### 3.5. Changes in Cardiac Size, Function, and NT-proBNP Following 2-Year Treatment for Predicting All-Cause Mortality/HTx

Each 5% annual decrease in LVEF (HR 2.066, *p* < 0.001), each 5 mm annual increase in LVD (HR 2.882, *p* < 0.001), each 5 mm annual increase in LAD (HR 2.531, *p* < 0.001), each 5 mm annual increase in RAD_short (HR 2.259, *p* = 0.002), each 5 mm annual increase in RAD_long (HR 1.938, *p* = 0.003), and each unit annual increase in Ln(NT-proBNP) (HR 3.366, *p* < 0.001) were independently associated with increased risk of all-cause mortality/HTx after adjusting for age, sex, BMI, etiology of DCM, and duration of treatment (Table 5).

In a multivariable Cox regression model that included these annual changes in LVEF, LVD, LAD, RAD_short, and NT-proBNP, along with age, sex, BMI, etiology, duration of treatment, smoking, and CRP, the analysis demonstrated that annual increases in LAD (HR 3.641, *p* < 0.001) and Ln(NT-proBNP) (HR 4.069, *p* < 0.001) were the strongest predictors of all-cause mortality/HTx, even after accounting for the effects of BMI (HR 0.857, *p* < 0.001), duration of treatment (HR 1.063, *p* = 0.031), and baseline CRP levels (HR 3.925, *p* = 0.002).

## 4. Discussion

Our cohort study of young adult patients with DCM identified several independent risk factors for all-cause mortality or HTx. The results indicate that smoking, digoxin use, elevated NT-proBNP levels (≥ 5678 pg/mL), higher CRP levels (≥ 3.0 mg/L), lower LVEF (≤ 25%), and enlarged LVD (≥ 65 mm) are significant risk factors for these adverse outcomes. Among these, elevated CRP and enlarged LVD were found to be the strongest independent risk factors. Moreover, our study underscores the importance of dynamic changes in cardiac parameters and the biomarker NT-proBNP level over time as predictors of all-cause mortality/HTx. Annual increases in LAD and NT-proBNP emerged as the strongest predictors of mortality and HTx. These findings highlight the critical role of ongoing monitoring of LAD and NT-proBNP levels in assessing the risk of adverse outcomes in young patients with DCM. Importantly, our study represents the first comprehensive clinical report to define these independent risk factors specifically for young adults with DCM, emphasizing the need for personalized monitoring and interventions to mitigate these risks and improve patient outcomes.

### 4.1. Prognostic Value of Smoking in Young Adult Patients With DCM

Both smoking and exposure to passive smoke are recognized as major, preventable contributors to cardiovascular morbidity and mortality [16]. Previous studies have shown that smoking and drinking are associated with poorer outcomes in patients with cardiomyopathy, highlighting their significant roles as risk factors for idiopathic congestive cardiomyopathy [17]. Data from New Zealand suggest that smoking is related to lower survival rates in DCM patients [18]. However, a few studies showed DCM patients who smoke might experience a more positive prognosis compared to nonsmokers [19]. Li et al. reported that neither smoking (HR 0.971, *p* = 0.663) nor drinking status (HR 0.891, *p* = 0.140) emerged as significant independent predictors of all-cause mortality in patients with DCM [20]. These findings underscore the unclear impact of smoking on DCM patients, highlighting the need for further scrutiny and interpretation of available data. In the present study, there was a notably higher prevalence of smoking in the nonsurvivor/HTx group (46.2% vs. 14.3%, *p* = 0.006), identifying smoking as an independent risk factor for all-cause mortality/HTx after adjusting for age, sex, and BMI. The discrepancies in findings regarding the association between smoking and prognosis in patients with DCM may arise from several factors. These include variations in study populations, differences in the definition and measurement of smoking status, varying durations of follow-up periods, and potential confounding variables that were not sufficiently addressed in prior studies. Additionally, the diversity in treatment strategies and adherence to smoking cessation interventions among different study cohorts could also contribute to the observed inconsistencies. These factors warrant further investigation and consideration to better understand the true impact of smoking on the prognosis of patients with DCM.

### 4.2. Prognostic Value of NT-proBNP and CRP in Young Adult Patients With DCM

Previous studies have demonstrated the prognostic significance of both NT-proBNP and CRP in cohorts with DCM [21–25]. Feng et al. compared these biomarkers between mildly DCM and advanced DCM subgroups. Their findings indicated that both NT-proBNP and high-sensitivity CRP independently correlated with adverse events across the DCM cohort. Interestingly, elevated high-sensitivity CRP levels specifically predicted poorer outcomes in mildly DCM patients [26]. In a clinical study by van der Meulen et al., NT-proBNP emerged as the singular independent predictor of adverse outcomes in both adult and pediatric DCM patients [3]. Consistent with these findings, our study demonstrated that every increment in the natural log-transformed NT-proBNP level triples the risk of experiencing either all-cause mortality or HTx. Future investigations should explore whether targeted medical interventions could enhance outcomes for these high-risk patients.

### 4.3. Prognostic Value of Serum UA in Young Adult Patients With DCM

The relationship between serum UA levels and DCM in adults has gained attention recently [27]. T. Li, H. Li, and Cheng found that elevated baseline serum UA levels in children with DCM were positively correlated with NYHA functional class and worse echocardiographic values. Moreover, they highlighted that dynamic changes in serum UA levels over time may serve as a biomarker reflecting the severity of DCM in this population [28]. In advanced DCM patients, Kim et al. [29] established UA as an independent predictor of prognosis, with concentrations exceeding or equal to 8.7 mg/dL associated with significantly decreased event-free survival. While reports on serum UA levels and outcomes in young adults with DCM are limited, our study addresses this gap. We observed that elevated serum UA levels in young adults with DCM were modestly associated with increased risks of all-cause mortality or HTx. UA levels ≥ 570 *μ*mol/L showed an HR of 2.016 (95% CI 0.964–4.217) for all-cause mortality or HTx, with a trend towards statistical significance (*p* = 0.063). Further research with larger sample sizes is needed to definitively establish the significance of this association.

### 4.4. Prognostic Value of LVEF and Cardiac Dimensions in Young Adult Patients With DCM

Echocardiography assessment is pivotal in the diagnosis, risk stratification, management, and follow-up of patients with DCM [30, 31]. In our cohort, we confirmed that an LVEF ≤ 25% and an LVD ≥ 65 mm were robust predictors of an increased risk for all-cause mortality or HTx, as determined by multivariate analysis. Additionally, our study provided compelling evidence that a 5% annual increase in LVEF was associated with a significant 35% reduction in the risk of all-cause mortality or HTx (HR 0.648, *p* = 0.024). Patients experiencing a 5 mm annual increase in LVD faced a twofold increase in the risk of adverse outcomes (HR 2.065, *p* = 0.009). Similarly, annual increases of 5 mm in LAD and RAD were independently associated with elevated risks of all-cause mortality or HTx. Notably, these associations remained significant even after adjusting for potential confounders such as age, sex, BMI, smoking, and digoxin use. These findings underscore the critical role of baseline and follow-up echocardiographic assessments in predicting adverse outcomes in young adult patients with DCM.

### 4.5. Clinical Implication

The findings of this study highlight critical factors in managing young adults with DCM. Smoking cessation, combined with adherence to guideline-directed medical therapy, is essential for improving outcomes. Regular monitoring of NT-proBNP and CRP levels can help identify high-risk patients early. Additionally, routine echocardiographic evaluations of LVEF and cardiac dimensions are crucial for detecting disease progression and adjusting treatments promptly. Addressing these risk factors holistically, considering elements like age, sex, BMI, smoking status, and medication use, can substantially enhance the management and prognosis of young adults with DCM.

### 4.6. Study Limitations

This study, conducted retrospectively at a single center, featured a relatively modest patient cohort. Despite our effort to include consecutive, homogeneous, and unselected patients with DCM, mirroring real-world scenarios, the small sample size prompts caution in generalizing the findings. A larger, more diverse cohort would undoubtedly bolster the robustness of our conclusions. Given the focus on young adults, the generalizability of our findings to older populations with DCM may be limited. Future research could explore age-specific patterns and outcomes.

## 5. Conclusions

In this study of young DCM patients aged 18–45 years, key prognostic factors for mortality and HTx included smoking, elevated NT-proBNP and CRP levels, lower LVEF (≤25%), and larger LVD (≥65 mm). Dynamic changes in LVEF, LVD, LAD, and RAD were also significant. The results add clinical evidence on risk factors for outcomes among young adults with DCM. Future studies are warranted to determine whether targeted medical care stratified by risk profile could improve outcomes in young DCM patients.

## Figures and Tables

**Table 1:** Baseline clinical and echocardiographic characteristics in young patients with DCM.

	**Total**	**Survivors**	**Nonsurvivors/HTx**	**p** ** value**
	**N** = 67	**N** = 28	**N** = 39	
Age (years)	31 ± 6	30 ± 5	32 ± 7	0.404
Male [*n* (%)]	50 (74.6)	22 (78.6)	28 (71.8)	0.530
BMI (kg/m^2^)	26.7 ± 6.1	29.5 ± 6.0	24.7 ± 5.3	0.001
Etiology				0.274
Idiopathic	31 (46.3)	13 (46.4)	18 (46.2)	
Familial/genetic	15 (22.4)	7 (25.0)	8 (20.5)	
Myocarditis	13 (19.4)	3 (10.7)	10 (25.6)	
Inflammatory/infectious	2 (3.0)	1 (3.6)	1 (2.6)	
Exposure to toxic substances	3 (4.5)	3 (10.7)	0 (0.0)	
Pregnancy-related	3 (4.5)	1 (3.6)	2 (5.1)	
Hypertension [*n* (%)]	33 (49.3)	12 (42.9)	21 (53.8)	0.375
Diabetes [*n* (%)]	1 (1.5)	1 (3.6)	0 (0.0)	1.000
Hypercholesterolemia [*n* (%)]	1 (1.5)	0 (0.0)	1 (2.6)	1.000
Chronic kidney disease [*n* (%)]	3 (4.5)	1 (3.6)	2 (5.1)	1.000
Smoking [*n* (%)]	22 (32.8)	4 (14.3)	18 (46.2)	0.006
Drinking [*n* (%)]	8 (11.9)	3 (10.7)	5 (12.8)	1.000
Medications [*n* (%)]				
Furosemide	66 (98.5)	28 (100)	38 (97.4)	1.000
Spironolactone	66 (98.5)	28 (100)	38 (97.4)	1.000
Sacubitril/valsartan	21 (31.3)	13 (46.4)	8 (20.5)	0.024
Beta-blockers	65 (97.0)	26 (92.9)	39 (100)	0.171
ACEIs	23 (34.3)	10 (35.7)	13 (33.3)	0.840
Digoxin	41 (61.2)	9 (32.1)	32 (82.1)	< 0.001
Laboratory data				
NT-proBNP (pg/mL)	6107 (2569–10526)	2321 (1328–5347)	9509 (5894–18091)	< 0.001
≥ 5678 pg/mL	36 (53.7)	6 (21.4)	30 (76.9)	< 0.001
cTnI (ng/mL)	0.02 (0.01–0.03)	0.02 (0.01–0.02)	0.02 (0.01–0.03)	0.151
CRP (mg/L)	3.37 (1.20–10.34)	1.20 (1.00–2.75)	6.00 (3.20–17.50)	< 0.001
≥ 3.0 mg/L	38 (56.7)	6 (21.4)	32 (82.1)	< 0.001
AST (U/L)	21.0 (17.0–32.0)	21.0 (17.0–43.5)	21.8 (18.0–32.0)	0.814
ALT (U/L)	21.0 (19.0–40.0)	20.5 (18.0–45.0)	23.0 (19.0–36.0)	0.688
SCr (*μ*mol/L)	77.0 (63.0–100.0)	77.0 (65.8–92.8)	78.9 (58.0–107.0)	0.904
TG (mmol/L)	1.23 (0.87–1.66)	1.35 (1.00–2.33)	1.13 (0.78–1.60)	0.100
TC (mmol/L)	4.23 (3.30–5.76)	4.40 (3.55–5.47)	4.10 (3.26–6.10)	0.909
LDL-C (mmol/L)	2.61 (2.00–3.48)	2.65 (2.00–3.38)	2.61 (1.80–3.60)	0.919
UA (*μ*mol/L)	667 (413–759)	523 (311–723)	682 (521–800)	0.012
≥ 570 *μ*mol/L	41 (61.2)	12 (42.9)	29 (74.4)	0.009
Echocardiography				
LVEF (%)	27.3 ± 5.9	30.3 ± 5.6	25.1 ± 5.1	<0.001
≤ 25%	46 (68.7)	12 (42.9)	34 (87.2)	<0.001
LVD (mm)	66.9 ± 6.2	64.3 ± 4.7	68.8 ± 6.5	0.003
≥ 65 mm	41 (61.2)	12 (42.9)	29 (74.4)	0.009
RVD (mm)	50.5 ± 15.2	50.9 ± 17.6	50.3 ± 13.5	0.873
LAD (mm)	50.7 ± 7.8	49.9 ± 8.0	51.4 ± 7.6	0.438
RAD_long (mm)	55.8 ± 9.1	53.3 ± 9.3	57.7 ± 8.5	0.050
RAD_short (mm)	43.4 ± 7.4	41.4 ± 7.3	44.9 ± 7.2	0.059

Abbreviations: ACEI, angiotensin-converting enzyme inhibitors; ALT, alanine aminotransferase; AST, aspartate aminotransferase; BMI, body mass index; CRP, C-reactive protein; DCM, dilated cardiomyopathy; HTx, heart transplantation; LAD, end-systolic left atrial diameter; LDL-C, low-density lipoprotein cholesterol; LVD, end-diastolic left ventricular diameter; LVEF, left ventricular ejection fraction; NT-proBNP, N-terminal pro-brain natriuretic peptide; RAD_long, end-systolic right atrial long-axis diameter; RAD_short, end-systolic right atrial short-axis diameter; RVD, end-diastolic right ventricular mid diameter; SCr, serum creatinine; TC, total cholesterol; TG, total triglyceride; UA, uric acid.

**Table 2:** Univariable Cox regression analysis identified the following potential risk factors of all-cause mortality/HTx.

	**HR (95% CI)** ^**a**^	**p** ** value**
Age (years)	1.022 (0.972–1.073)	0.400
Male [*n* (%)]	0.650 (0.321–1.320)	0.233
BMI (kg/m^2^)	0.905 (0.853–0.960)	0.001
Smoking [*n* (%)]	2.101 (1.106–3.990)	0.023
Sacubitril/valsartan	0.463 (0.212–1.011)	0.053
Digoxin	3.294 (1.446–7.491)	0.005
Ln(NT-proBNP)	2.045 (1.420–2.945)	< 0.001
≥ 5678 pg/mL	2.876 (1.365–6.060)	0.005
Ln(CRP)	1.512 (1.201–1.904)	< 0.001
≥ 3.0 mg/L	5.051 (2.221–11.489)	< 0.001
UA (*μ*mol/L)	1.002 (1.000–1.003)	0.029
≥ 570 *μ*mol/L	2.020 (0.984–4.150)	0.056
LVEF (%)	0.891 (0.837–0.948)	< 0.001
≤ 25%	2.738 (1.415–5.296)	0.003
LVD (mm)	1.044 (1.005–1.084)	0.025
≥ 65 mm	2.179 (1.053–4.510)	0.036
RAD_long (mm)	1.028 (0.993–1.064)	0.116

Abbreviations: BMI, body mass index; CI, confidence interval; CRP, C-reactive protein; HR, hazard ratio; HTx, heart transplantation; Ln, natural logarithm transformation; LVD, end-diastolic left ventricular diameter; LVEF, left ventricular ejection fraction; NT-proBNP, N-terminal pro-brain natriuretic peptide; RAD_long, end-systolic right atrial long-axis diameter; UA, uric acid.

^a^The follow-up period spanned from the baseline visit to the endpoint or the last day of follow-up.

**Table 3:** Changes in cardiac function and chambers and NT-proBNP levels following 2-year treatment and their prognostic performance for all-cause mortality/HTx in young patients with DCM.

	**T0**	**T1**	**p** ** value**	**Annual difference**	**Each 1 unit difference/year HR (95% CI)** ^**a**^	**p** ** value**
	**Median duration of 2 (1–2) years**				**Median duration of 3 (1–4) years**	
LVEF (%)	27 (22–31)	30 (22–36)	0.026	0.50 (−1.50 to 4.33)	Each 1% annual decrease 1.088 (1.028–1.152)	0.004
Survivors	31 (26–35)	39 (33–44)	< 0.001	5.00 (0.63–7.88)		
Nonsurvivors/HTx	25 (21–29)	23 (20–30)	0.266	−0.33 (−2.50 to 1.00)		
*p* value				< 0.001		
LVD (mm)	67 (62–71)	69 (60–74)	0.354	1.67 (−1.50 to 3.00)	Each 1 mm annual increase at 1.229 (1.110–1.361)	< 0.001
Survivors	63 (60–68)	60 (53–67)	0.007	−2.75 (−8.25 to 2.88)		
Nonsurvivors/HTx	68 (63–73)	72 (70–76)	< 0.001	2.00 (1.00–4.00)		
*p* value				< 0.001		
RVD (mm)	49 (38–65)	55 (44–65)	0.829	1.00 (−1.00 to 4.00)	Each 1 mm annual increase at 1.025 (0.986–1.066)	0.205
Survivors	50 (33–68)	53 (40–61)	0.441	−0.50 (−4.25 to 2.00)		
Nonsurvivors/HTx	49 (40–61)	56 (45–68)	< 0.001	2.00 (0.50–4.00)		
*p* value				0.014		
LAD (mm)	52 (46–54)	52 (45–57)	0.330	1.00 (−2.00 to 2.00)	Each 1 mm annual increase at 1.077 (1.005–1.154)	0.037
Survivors	51 (45–55)	46 (43–52)	0.107	−1.50 (−3.88 to 2.00)		
Nonsurvivors/HTx	52 (48–54)	54 (49–57)	0.001	2.00 (0.00–2.50)		
*p* value				0.005		
RAD_long (mm)	56 (50–61)	58 (48–64)	0.017	0.50 (−2.50 to 3.00)	Each 1 mm annual increase at 1.087 (1.021–1.157)	0.009
Survivors	53 (46–60)	48 (45–53)	0.015	−2.25 (−5.75 to 0.38)		
Nonsurvivors/HTx	57 (52–61)	60 (57–68)	< 0.001	2.00 (0.00–3.50)		
*p* value				< 0.001		
RAD_short (mm)	43 (38–49)	42 (38–50)	0.193	0.50 (−2.00 to 2.00)	Each 1 mm annual increase at 1.172 (1.058–1.298)	0.002
Survivors	41 (36–46)	39 (34–54)	0.014	−1.00 (−3.88 to 2.00)		
Nonsurvivors/HTx	45 (40–49)	48 (42–52)	0.012	0.50 (−0.50 to 2.00)		
*p* value				0.001		
NT-proBNP (pg/mL)	6107 (2569–10526)	3180 (1002–8187)	< 0.001	−967 (−3610 to 75)	Each 1 unit annual increase at 2.896^b^ (1.686–4.975)	< 0.001
Survivors	2321 (13228–5347)	861 (389–1284)	< 0.001	−1024 (−3391 to −418)		
Nonsurvivors/HTx	9509 (5894–18091)	7068 (3539–12078)	0.018	−862 (−3610 to 446)		
*p* value				< 0.001		

Abbreviations: CI, confidence interval; DCM, dilated cardiomyopathy; HR, hazard ratio; HTx, heart transplantation; LAD, end-systolic left atrial diameter; LVD, end-diastolic left ventricular diameter; LVEF, left ventricular ejection fraction; NT-proBNP, N-terminal pro-brain natriuretic peptide; RAD_long, end-systolic right atrial long-axis diameter; RAD_short, end-systolic right atrial short-axis diameter; RVD, end-diastolic right ventricular mid diameter.

^a^The follow-up period spanned from the completion of the 2-year medical therapy to the endpoint or the last day of follow-up.

^b^Ln (natural logarithm) transformed.

**Table 4:** Baseline clinical and echocardiographic factors for all-cause mortality/HTx in young patients with DCM.

	**Age, sex, BMI, etiology, and duration of treatment adjusted Cox model** ^**a**^	**p** ** value**
	**HR (95% CI)**	

Smoking	2.339 (1.251–4.600)	0.008
Sacubitril/valsartan	0.650 (0.287–1.473)	0.302
Digoxin	2.535 (1.089–5.901)	0.031
Ln(NT-proBNP)	1.959 (1.334–2.876)	< 0.001
NT-proBNP ≥ 5678 pg/mL	2.284 (1.072–4.863)	0.032
Ln(CRP)	1.672 (1.305–2.142)	< 0.001
CRP ≥ 3.0 mg/L	5.654 (2.447–13.067)	< 0.001
UA	1.002 (1.000–1.004)	0.018
UA ≥ 570 *μ*mol/L	1.928 (0.938–3.964)	0.074
LVEF (%)	0.908 (0.855–0.966)	0.002
LVEF ≤ 25%	2.370 (1.216–4.619)	0.011
LVD (mm)	1.074 (1.028–1.121)	0.001
LVD ≥ 65 mm	2.110 (1.016–4.380)	0.045

	**Multivariable model including smoking, digoxin, NT-proBNP ≥ 5678 pg/mL, CRP ≥ 3.0 mg/L, UA ≥ 570 *μ*mol/L, LVEF ≤ 25%, and LVD ≥ 65 mm, along with age, sex, BMI, etiology, and duration of treatment** ^**b**^	**p** ** value**
	**HR (95% CI)**	

Male	0.320 (0.134–0.765)	0.010
BMI	0.904 (0.848–0.965)	0.002
Smoking	1.915 (0.895–4.100)	0.094
CRP ≥ 3.0 mg/L	6.727 (2.786–16.241)	< 0.001
LVEF ≤ 25%	1.931 (0.938–3.975)	0.074
LVD ≥ 65 mm	3.038 (1.360–6.789)	0.007

Abbreviations: CI, confidence interval; DCM, dilated cardiomyopathy; HR, hazard ratio; HTx, heart transplantation; LAD, end-systolic left atrial diameter; Ln, natural logarithm transformation; LVD, end-diastolic left ventricular diameter; LVEF, left ventricular ejection fraction; NT-proBNP, N-terminal pro-brain natriuretic peptide; RAD_long, end-systolic right atrial long-axis diameter; RAD_short, end-systolic right atrial short-axis diameter; RVD, end-diastolic right ventricular mid diameter.

^a^Each parameter that is associated with all-cause mortality/HTx identified by univariable Cox regression (*p* < 0.10 in Table 2) was adjusted for age, sex, BMI, etiology, and duration of treatment with a backward stepwise (likelihood ratio) method, respectively. The follow-up period spanned from the baseline visit to the endpoint or the last day of follow-up.

^b^All parameters were entered into a single model with a backward stepwise (likelihood ratio) method. The follow-up period spanned from the baseline visit to the endpoint or the last day of follow-up.

**Table 5:** Changes in cardiac size, function, and NT-proBNP following a 2-year treatment for predicting all-cause mortality/HTx in young patients with DCM.

	**Age, sex, BMI, etiology, and duration of treatment adjusted Cox model** ^**a**^	**p** ** value**
	**HR (95% CI)**	

Each 5% annual decrease in LVEF	2.066 (1.357–3.146)	< 0.001
Each 5 mm annual increase in LVD	2.882 (1.585–5.240)	< 0.001
Each 5 mm annual increase in LAD	2.531 (1.472–4.353)	< 0.001
Each 5 mm annual increase in RAD_long	1.938 (1.260–2.981)	0.003
Each 5 mm annual increase in RAD_short	2.259 (1.361–3.750)	0.002
Each unit annual increase in Ln(NT-proBNP)	3.366 (1.685–6.724)	< 0.001

	**Multivariable Cox model including annual changes in LVEF, LVD, LAD, RAD_short, and Ln(NT-proBNP), along with age, sex, BMI, etiology, duration of treatment, smoking, and CRP ≥ 3.0 mg/L** ^**b**^	**p** ** value**
	**HR (95% CI)**	

BMI	0.857 (0.795–0.925)	< 0.001
Duration of treatment	1.063 (1.006–1.123)	0.031
CRP ≥ 3.0 mg/L	3.925 (1.673–9.210)	0.002
Each 5 mm annual increase in LAD	3.641 (1.699–7.804)	< 0.001
Each unit annual increase in Ln(NT-proBNP)	4.069 (1.843–8.984)	< 0.001

Abbreviations: CI, confidence interval; DCM, dilated cardiomyopathy; HR, hazard ratio; HTx, heart transplantation; LAD, end-systolic left atrial diameter; Ln, natural logarithm transformation; LVD, end-diastolic left ventricular diameter; LVEF, left ventricular ejection fraction; NT-proBNP, N-terminal pro-brain natriuretic peptide; RAD_long, end-systolic right atrial long-axis diameter; RAD_short, end-systolic right atrial short-axis diameter; RVD, end-diastolic right ventricular mid diameter.

^a^Each parameter that is associated with all-cause mortality/HTx identified by univariable Cox regression (*p* < 0.10 in Table 3) was, respectively, adjusted for age, sex, BMI, etiology, and duration of treatment with a backward stepwise (likelihood ratio) method. The follow-up period spanned from the completion of the 2-year medical therapy to the endpoint or the last day of follow-up.

^b^All parameters were entered into a single model with a backward stepwise (likelihood ratio) method. The follow-up period spanned from the completion of the 2-year medical therapy to the endpoint or the last day of follow-up.

## Data Availability

Data are available upon reasonable request (contact the corresponding author Dr. Junhua Ge).

## Nomenclature

ACEI: angiotensin-converting enzyme inhibitors
ALT: alanine aminotransferase
AST: aspartate aminotransferase
BMI: body mass index
CI: confidence interval
SCr: serum creatinine
CRP: C-reactive protein
DCM: dilated cardiomyopathy
HR: hazard ratio
HTx: heart transplantation
LAD: end-systolic left atrial diameter
LDL-C: low-density lipoprotein cholesterol
LVEF: left ventricular ejection fraction
LVD: end-diastolic left ventricular diameter
NT-proBNP: N-terminal pro-brain natriuretic peptide
RAD_long: end-systolic right atrial long-axis diameter
RAD_short: end-systolic right atrial short-axis diameter
RVD: end-diastolic right ventricular mid diameter
TC: total cholesterol
TG: triglyceride
UA: uric acid
